# Preparation of PSF/FEP mixed matrix membrane with super hydrophobic surface for efficient water-in-oil emulsion separation†

**DOI:** 10.1039/c8ra00055g

**Published:** 2018-03-14

**Authors:** Dawei Ji, Changfa Xiao, Shulin An, Hailiang Liu, Kaikai Chen, Junqiang Hao, Tai Zhang

**Affiliations:** State Key Laboratory of Separation Membranes and Membrane Processes, National Center for International Joint Research on Separation Membranes, School of Material Science and Engineering, Tianjin Polytechnic University Tianjin 300387 China xiaochangfa@163.com

## Abstract

Polysulfone (PSF)/fluorinated ethylene propylene (FEP) mixed matrix membranes (MMMs) with super hydrophobic surface were successfully fabricated *via* non-solvent induced phase separation (NIPS) method. The effects of FEP content on the morphology, roughness, wettability, pore size, and mechanical property of PSF/FEP MMMs were characterized by scanning electron microscope, confocal microscopy, contact angle goniometer, mercury porosimetry, and tensile testing instrument, respectively. When the FEP content was 9 wt%, the average roughness of M-4 reached 0.712 μm. Meanwhile, the water contact angle (CA) and the water sliding angle (SA) was 153.3° and 6.1°, respectively. M-4 showed super hydrophobicity with a micro- and nanoscale structure surface. Then, M-4 was used for separating of water-in-oil emulsion, showing high separation efficiency for water-in-kerosene and water-in-diesel emulsions of 99.79% and 99.47%, respectively. The flux and separation efficiency changed slightly after 10 cycles. Therefore, this study indicated that the obtained PSF/FEP MMM with super hydrophobic surface could be used for efficient water-in-oil emulsion separation.

## Abstract 2

The PSF/FEP membrane with super hydrophobic and super oleophilic surface had an outstanding separation performance for water-in-oil emulsion.

## Graphical abstract

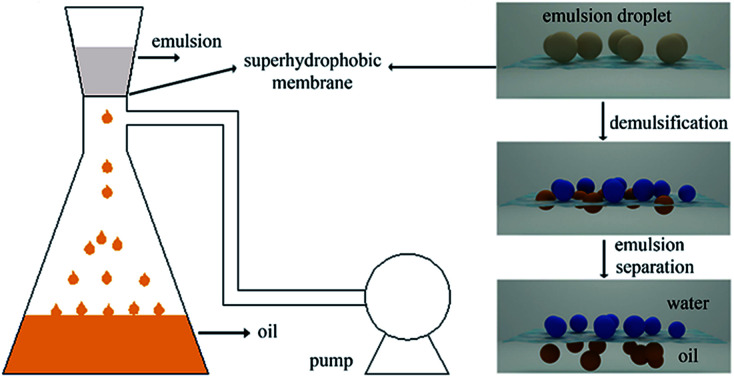

## 1. Introduction

Environmental pollution has got increasingly serious with the rapid expansion of industrialization. Emulsified oil/water mixtures are generated in diverse industries, such as petrochemical, food, textile, leather, steel, aluminum, and metal finishing, the direct discharge of which will threaten the environment and human health.^1,2^Separating water from emulsified oil/water mixtures is extremely important for the treatment of water-in-oil emulsion and the purification of oil, such as fuel oil, automotive oil, and transformer oil. The separation of emulsified oil/water mixtures is always difficult and challenging because the droplets are usually stable and nanoscale.^3^ Conventional techniques such as skimming, ultrasonic separation, air flotation, gravity processing, coagulation–flocculation, and chemical de-emulsification method are often limited by their low separation efficiency, high energy cost, complex separation devices and secondary pollution *etc.*^4–6^ Very recently, membrane filtration has become an important technology in the oil/water separation field because of its many advantages, such as high filtration efficiency, great selectivity, low energy cost, and environmental friendly.^7–9^

Generally, the membranes are classified as the hydrophobic–oleophilic membrane and hydrophilic–oleophobic membrane for “oil-removing” and “water-removing” process, respectively.^10^ A series of “oil-removing” membranes have been fabricated for highly efficient oil/water separation by designing superhydrophobic and superoleophilic surfaces. Li *et al.*^11^ immobilized Ag nanocluster on nano-fibrous membrane for oil/water separation. Cao *et al.*^12^ modified stainless steel mesh using chemistry and Michael addition reaction by *n*-dodecyl mercaptan for efficient oil/water separation. Zhang *et al.*^13^ fabricated superhydrophobic and superoleophilic polyester materials by one-step growth of silicone nanofilaments onto the textile *via* chemical vapor deposition of trichloromethylsilane for oil/water separation. These materials usually show ultrahigh permeation flux for the separation of immiscible oil/water mixtures. Nevertheless, these materials could not effectively separate emulsified oil/water mixtures because their pore sizes (about tens of micrometers) are much larger than the droplet sizes of the emulsion (typically less than 20 μm).^14^

Superhydrophobic surface is one of the most important parameters for the separation of water-in-oil emulsion. When emulsion droplets touch the superhydrophobic surface, a de-emulsification process could proceed immediately on the membrane surface due to the different wettability for oil and water.^2^ Meanwhile, the oil quickly penetrates through the membrane and water is retained above. Generally, the emulsion separation efficiency increases with the improvement of hydrophobicity.^15^

The preparation of the superhydrophobic membranes for the separation of water-in-oil emulsion usually requires expensive materials, strict conditions (such as harsh chemical treatment), and complex processing methods including plasma etching, chemical vapor deposition, electrodeposition, calcination and the use of templates,^11,13,16–20^ which can be challenging for the large-scale membrane fabrication.

PSF is a classical membrane material for the preparation of ultrafiltration,^21–24^ nanofiltration,^25,26^ and matrix membranes^27–29^ due to its outstanding properties of thermal stability, chemical resistance, high mechanical strength, and wide aperture adjustment range.^30^ Hydrophobic SiO_2_ nanoparticles have the features of small particle size, narrow particle size distribution, and large surface area,^31^ which play an important role in making a rough and superhydrophobic surface.^15,32–34^ FEP resin has lower surface free energy due to its chemical compositions, which is usually used to prepare and modify for hydrophobic membrane.^35–37^

Herein, we report the fabrication of PSF/FEP mixed matrix membranes (MMMs) for water-in-oil emulsion separation by using environmentally friendly and low cost chemicals as well as under simple and easy conditions to meet the need on an industrial scale. SiO_2_ nanoparticles and micro-nanometer scale FEP particles were introduced into PSF casting solution to fabricate micro- and nanoscale hierarchical structures on the membrane surface. Meanwhile, the surface energy of membrane decreased due to the existing of FEP on the surface, which was beneficial to fabricating of superhydrophobic surface, and there were obvious improvements on the comprehensive performance of the as-prepared MMMs. The effects of FEP content on MMMs' performances in terms of hydrophobicity, mechanical strength, permeability, and separation performance of water-in-oil emulsion were investigated respectively.

## 2. Experimental

### 2.1 Materials

Polysulfone (PSF) was purchased by Solvay Group-Solvay (Shanghai) Co., Ltd. Poly(tetrafluoroethylene-*co*-hexafluoropropylene) resin (6100) was purchased from DuPont Co., Ltd (U.S.A.). Hydrophobicity SiO_2_ particles (average particle size 40 nm) were purchased from Guangzhou GBS High-tech & Industry Co., Ltd. *N*,*N*-Dimethylacetimide (DMAc, 98%) was obtained from Samsung Fine Chemical Co., Ltd. (Ulsan, Korea). Di-*n*-octylo-phthalate (DOP), ethyl alcohol, Span80 (Hydrophile–Lipophile Balance (HLB) = 4.3), kerosene and diesel were bought from Tianjin Fengchuan Chemical Reagent Technologies Co. Ltd.

### 2.2 Membrane preparation

Before using, PSF, FEP resin and SiO_2_ particles were dried in an oven at 80 °C for 12 h to wipe out the residual moisture. In the process of preparing casting solution, DOP and DMAc were mixed homogeneously at 80 °C for 30 min; then, SiO_2_ particles were introduced into the mixed stirring for 1 h and ultrasound 30 min to guarantee SiO_2_ particles homogeneous dispersion. PSF was dissolved in the mixed for 3 h at 80 °C. Finally, FEP was introduced into the previous solution stirring for 3 h. After degassing for 30 min with atmospheric pressure, the casting solution was casted into flat sheet membranes on the clean and smooth glass substrate. Then, the membranes were immersed into the deionized water for 5 min and soaked in the ethyl alcohol for 24 h to remove solvent and additive. Finally, membranes with different FEP content were obtained and dried in the air for the analytical test. The as-prepared membranes with different FEP content were labeled as M-1, M-2, M-3, M-4, and M-5, respectively. The composition of PSF/FEP MMMs were listed in Table 1.

**Table 1:** The composition of PSF/FEP MMMs

Membrane	PSF (wt%)	FEP (wt%)	SiO_2_ (wt%)	DOP (wt%)	DMAc (wt%)
M-1	9	0	3	30	58
M-2	9	3	3	30	55
M-3	9	6	3	30	52
M-4	9	9	3	30	49
M-5	9	12	3	30	46

### 2.3 Characterization of PSF/FEP MMMs

PSF/FEP MMMs had large roughness, which was not suitable for quantifying by Atomic Force Microscope (AFM). So, surface roughness of the membrane was measured by Confocal Scanning Microscope (CSM700, Zeiss, Germany). The samples were cut into 5 mm (width) × 5 mm (length) test strips and then were stick on the smooth glass. The morphologies of membrane surface and cross-section were observed using field emission scanning electron microscopy (FESEM, S4800, HITACHI Japan). The samples were coated in gold before testing. The mechanical properties of PSF/FEP MMMs were measured by YG-061-1500 tensile tester at room temperature. The samples were cut into 5 mm (width) × 50 mm (length) test strips. Each sample was measured 5 times at the tensile rate of 2 mm min^−1^. The wettability of PSF/FEP MMMs were measured *via* Optical contact angle meter (JYSP-180, Jinshengxin Inspection instrument Co., Ltd.) by measuring the contact angle of water and oil. The water droplet and oil droplet about 2 μl were dropped on the membrane surface, respectively. The porosity of PSF/FEP MMMs were measured by the gravimetric method. The porosity (*ε*) was used to calculate by the following eqn (1):1
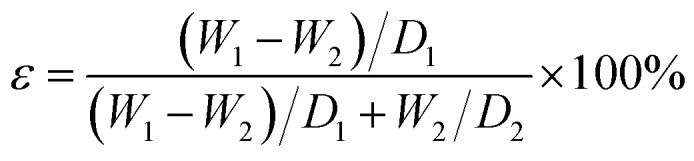
where, *W*_1_ is the weight of the wet membrane, *W*_2_ is the weight of the dry membrane, *D*_1_ is the *n*-butyl alcohol density, and *D*_2_ is the density of PSF/FEP MMMs.

The pore size and pore size distribution of membranes were measured by mercury porosimetry (Autopore IV9500, Tektronix, USA). The oil flux of PSF/FEP MMMs were measured by a laboratory device (Fig. 11(a)). The samples were located between the feed liquid bottle and the filter tip sealed by PTFE tape carefully. The oil flux was measured at −0.09 MPa and the flux was calculated by the following eqn (2):2
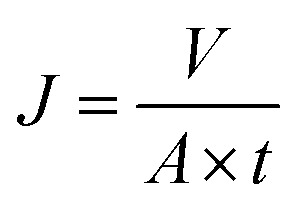
where, *J* is the oil flux (L m^−2^ h^−1^), *V* is the oil total volume (L), *A* is the membrane area (m^2^), *t* is the operating time (h).

### 2.4 Emulsion separation experiment

Surfactant-stabilized water-in-oil emulsion is stable and difficult to separate. In this work, we selected surfactant-stabilized water-in-oil emulsion to test the separating performance of PSF/FEP MMMs. Surfactant-stabilized water-in-oil emulsions were prepared by adding 0.5 g Span80 into 100 g oil (kerosene and diesel), stirring 30 min, then adding 5 g water dropwise slowly, and vigorously stirring for more than 3 h. The water-in-oil emulsions of kerosene and diesel were stable for separation experiment. The separation experiment was conducted by the laboratory device as shown in Fig. 11(a). The fresh water-in-oil emulsions were separated through PSF/FEP MMMs under a negative pressure (−0.09 MPa). The water content of fresh water-in-oil emulsions and collected filtrate were analyzed using a Karl Fischer titrator. For each PSF/FEP MMM, five measurements were tested to obtain an average value. The separation efficiency was calculated by the following eqn (3):3
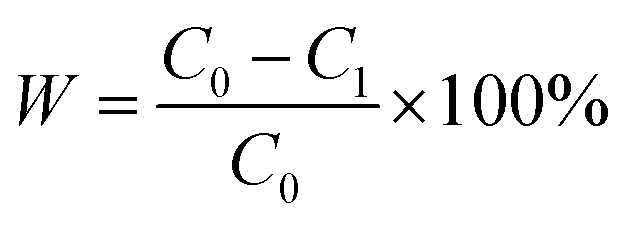
where, *W* is the separation efficiency (%), *C*_0_ is the water content of fresh water-in-oil emulsions (ppm), *C*_1_ is the water content of collected filtrate (ppm).

## 3. Results and discussion

### 3.1 Surface roughness

**Fig. 1:**
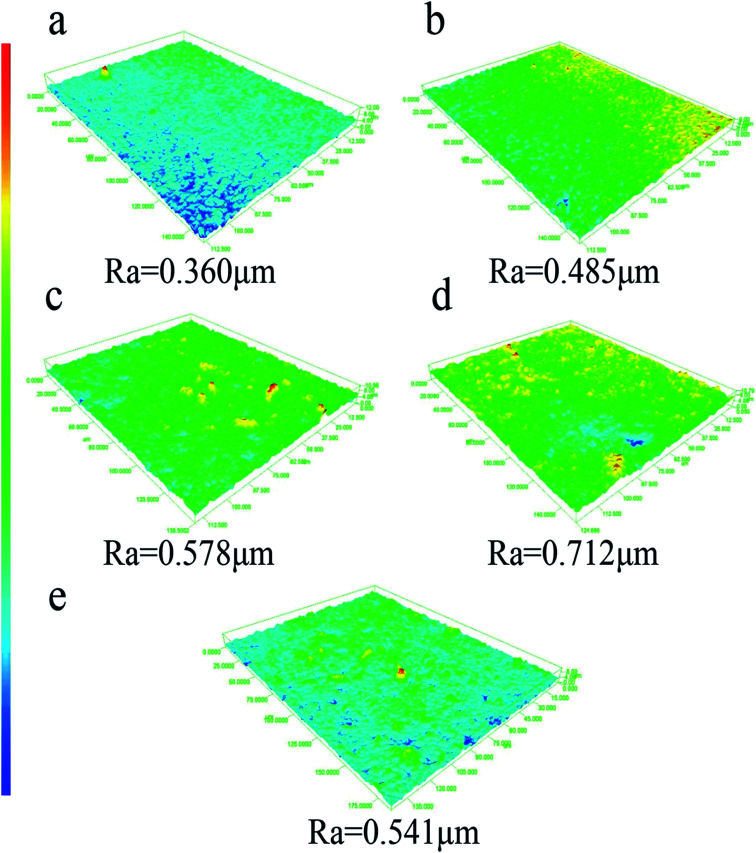
Surface roughness images of PSF/FEP MMMs with different FEP contents. (a) M-1, (b) M-2, (c) M-3, (d) M-4 and (e) M-5.

Conventionally, super hydrophobic surface has been produced mainly by two ways.^12^ One is to create a rough structure on the hydrophobic surface,^38^ and the other is to modify a rough surface by low surface free energy materials.^39^ However, it has demonstrated that the –CF_3_– terminated surface possessed the lowest free energy, while the maximum water contact angle could only reach about 120° on flat surface.^40^ As described in Wenzel equation,^41^ it could be known that the surface roughness was also a key factor for super hydrophobic surface. The membrane surface roughness was showed in Fig. 1. The image colors represented different surface roughness size, red and blue were on behalf of large and small surface roughness, respectively. When the content of FEP was 9 wt%, the surface roughness was the largest of 0.712 μm because of forming micro- and nanoscale hierarchical structures on the membrane surface. Furthermore, micro- and nanoscale structures of the membrane surface were destroyed due to redundant FEP (12 wt%), so the surface roughness decreased.

### 3.2 Morphology

**Fig. 2:**
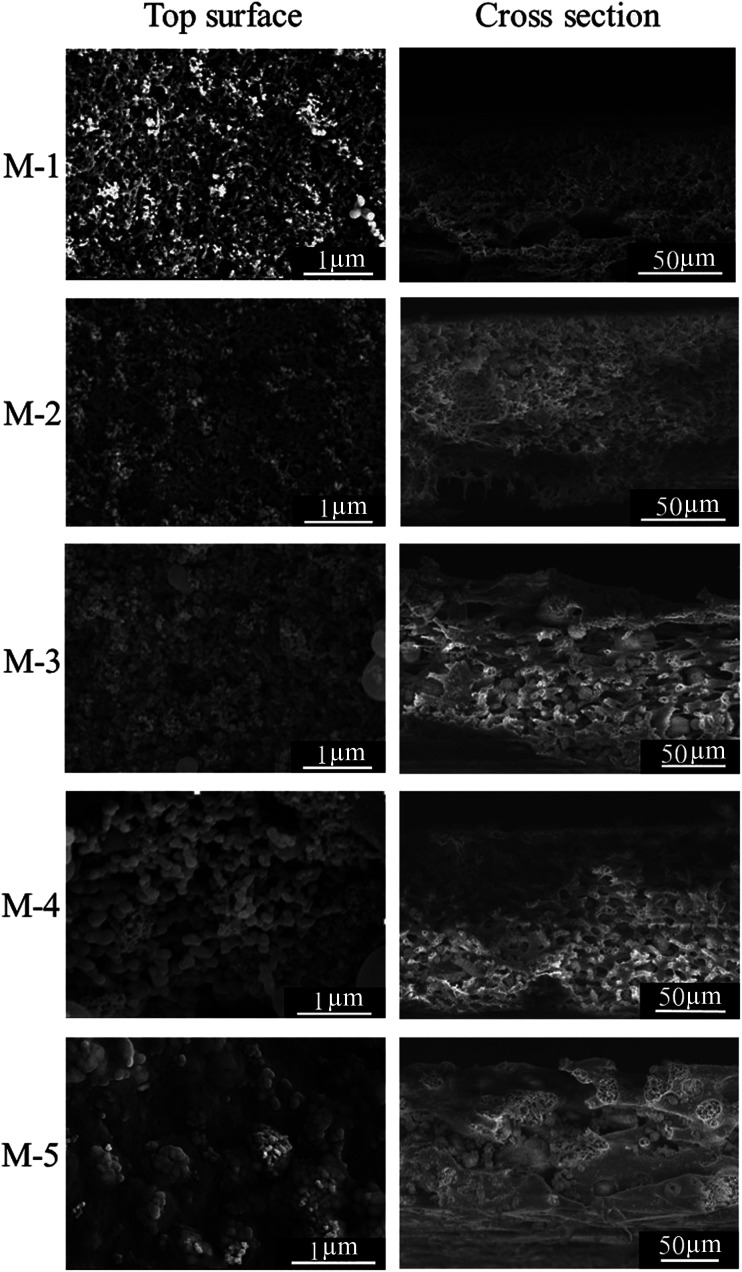
FESEM images of PSF/FEP MMMs with different FEP contents.

Morphologies of PSF/FEP MMMs with different FEP contents were observed by FESEM. As shown in Fig. 2, there were many papillae on the membrane surface with the addition of SiO_2_ nanoparticles and micro-nanometer scale FEP particles. When the FEP content was 9 wt%, many papillae were clearly found on the M-4 surface with average diameter about 145 nm. These micro- and nanoscale structures improved the membrane surface roughness and hydrophobicity. However, when the content of FEP was up to 12 wt%, FEP particles were inclined to aggregate, which destroyed the structures of membrane surface. Morphologies of the membrane cross-sections were shown in Fig. 2, the cross-sections presented sponge-like pore structure, and there were some heterogeneous macro pores in the cross-sections. With increasing FEP concentration, the cross-section pore structure got bigger. When the FEP concentration continued to increase to 12 wt%, an excess of FEP particles blocked the phase separation of matrix PSF and dispersion phase FEP, leading to destroying the pore structures.

The thickness of PSF/FEP MMMs was shown in Table 2. It could be found that the thickness improved obviously due to the improvement of the solid content with increasing FEP concentration.

**Table 2:** The properties of PSF/FEP MMMs

Membrane	M-1	M-2	M-3	M-4	M-5
Roughness (μm)	0.360	0.485	0.578	0.712	0.541
Thickness (μm)	97.65	106.34	131.02	132.97	140.59
Tensile strength (MPa)	1.40	1.51	1.67	1.88	1.03
Elongation at break (%)	22.32	8.58	10.14	11.16	8.56
Water contact angle (°)	110.8	121.2	131.3	153.3	124.0
Water sliding angle (°)	—	—	—	6.1	—
Pore size (nm)	116.2	160.3	167.9	165.3	171.5
Porosity (%)	58.9	61.3	66.5	74.3	52.3
Flux of kerosene (L m^−2^ h^−1^)	176.8	183.9	267.2	352.4	112.3
Flux of diesel (L m^−2^ h^−1^)	329.6	357.8	516.5	668.8	214.2
Rejection of water-in-kerosene (%)	—	—	89.94	99.79	94.92
Rejection of water-in-diesel (%)	—	—	88.36	99.47	93.87

### 3.3 Mechanical strength

**Fig. 3:**
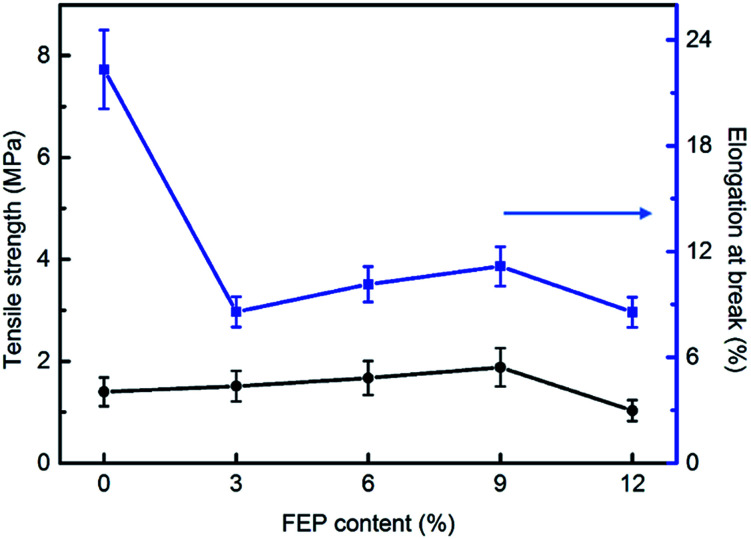
Mechanical properties of PSF/FEP MMMs.

The mechanical properties of PSF/FEP MMMs were discussed and shown in Fig. 3. For M-1, the elongation at break was the biggest of 22.06%, while tensile strength was low. With increasing FEP concentration, FEP particles changed the intrinsic structure of PSF matrix, the elongation at break decreased obviously. When the content of FEP was 9 wt%, the PSF/FEP membrane had good elongation at break of 11.1% and the biggest tensile strength. The main reason was that M-4 had homogeneous cross-section pore structure, as shown in Fig. 2, which improved the tensile strength and the elongation at break. When FEP concentration was up to 12 wt%, the membrane structure was destroyed resulting in the tensile strength and elongation at break decreasing obviously.

### 3.4 Wettability of the membrane surface

**Fig. 4:**
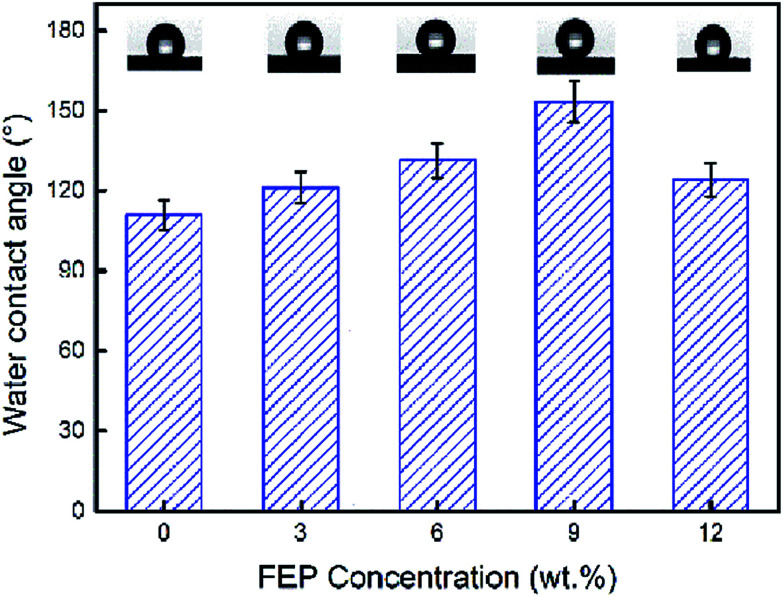
Water contact angle of PSF/FEP MMMs with different FEP concentration.

**Fig. 5:**
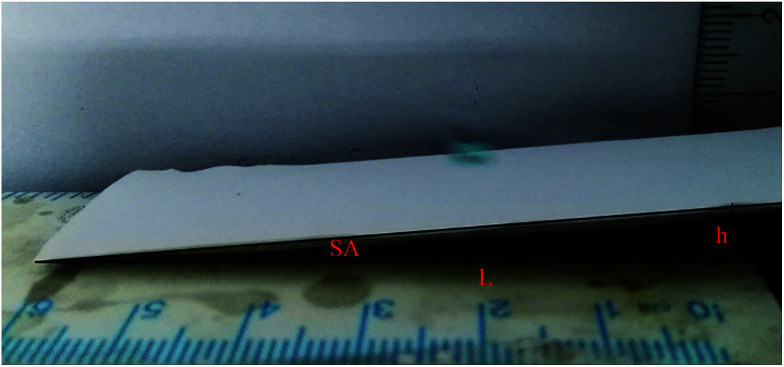
The instantaneous sliding behavior of a water droplet (dyed with methylene blue) on the M-4 surface.

Super hydrophobic and super oleophilic membrane surface is one of the most important properties for the efficient separation of water-in-oil emulsion. Generally, surfaces with a water contact angle (CA) larger than 150° and a water sliding angle (SA) lower than 10° are considered to be super hydrophobic surfaces.^32^Fig. 4 showed the CA changing from 110.8° to 153.3°, as the content of FEP increased from 0 wt% to 12 wt% with constant 3 wt% SiO_2_. When FEP concentration was 9 wt%, the micro- and nanoscale hierarchical structures were fabricated on the M-4 surface. The CA of M-4 was about 153.3°. And the SA of M-4 was about 6.1° lower than 10° (calculated by the equation of SA = arctan (h L^−1^)) determined by placing a water droplet on its surface, which was inclined to tilt gradually until water droplet started to roll, as shown in Fig. 5. The result indicated that M-4 exhibited super hydrophobicity. When FEP concentration was 12 wt%, the hierarchical structures of the membrane surface were destroyed and the surface roughness decreased obviously, ultimately resulting in the decrease of hydrophobicity.

**Fig. 6:**
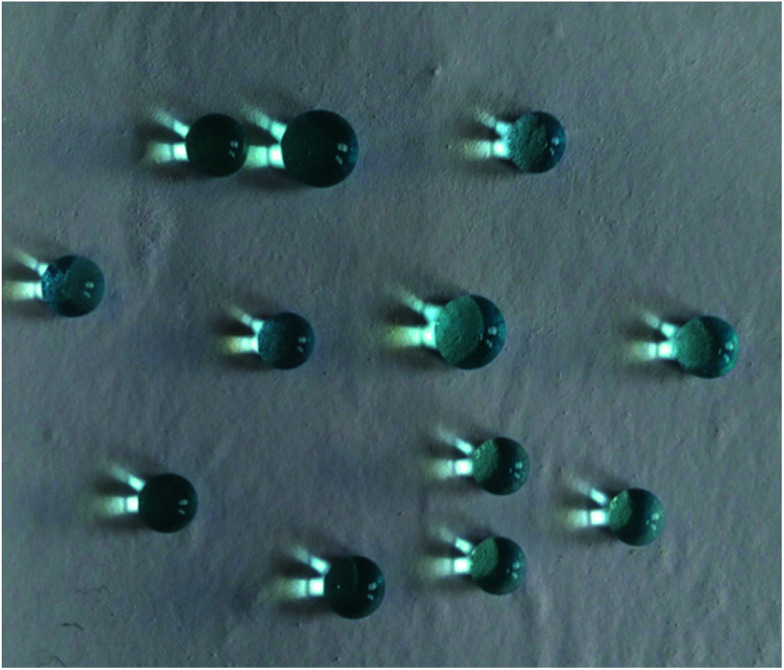
Photograph of water droplets (dyed with methylene blue) on the M-4 surface.

As shown in Fig. 6, the water droplets (dyed with methylene blue) kept sphere without infiltrating the membrane surface, which demonstrated the super hydrophobicity of M-4.

**Fig. 7:**
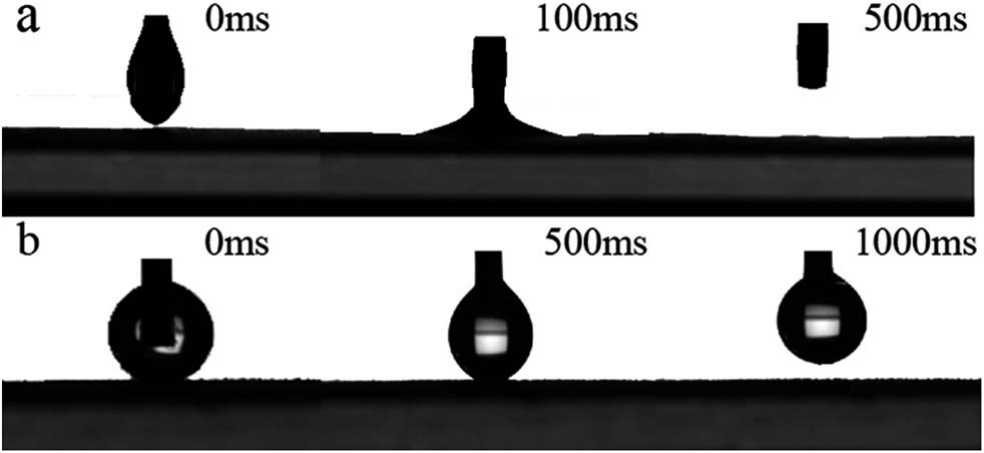
Variation of kerosene (a) and water (b) contact angle depended on time for M-4 surface.

The oil contact angle of PSF/FEP MMM was tested using kerosene on the M-4 surface. As shown in Fig. 7(a), the oil (kerosene) droplet was squeezed onto M-4 surface, then the oil droplet stuck to the membrane surface fast within 100 ms; after-wards, the needle was withdrew, the kerosene droplet rapidly spread out on the membrane surface and disappeared within 500 ms. The membrane exhibited good oleophilicity.

Furthermore, the water-adhesion property of M-4 surface was also characterized. Fig. 7(b) showed the process of water droplet on the membrane surface. When the needle approaching M-4 surface, the water droplet was squeezed onto the surface, then the water droplet was forced to sufficiently contact the membrane surface with an obvious deformation; after-wards, the needle was withdrew, the water droplet stuck completely to the needle and no trace of water was detected on the membrane surface. That was because the M-4 surface was rough and hydrophobic, which trapped water molecules in a Wenzel state. Meanwhile, the contact area decreased between water droplet and the membrane surface. The result further indicated that M-4 exhibited super hydrophobicity and super oleophilicity.

### 3.5 Porosity and pore size

**Fig. 8:**
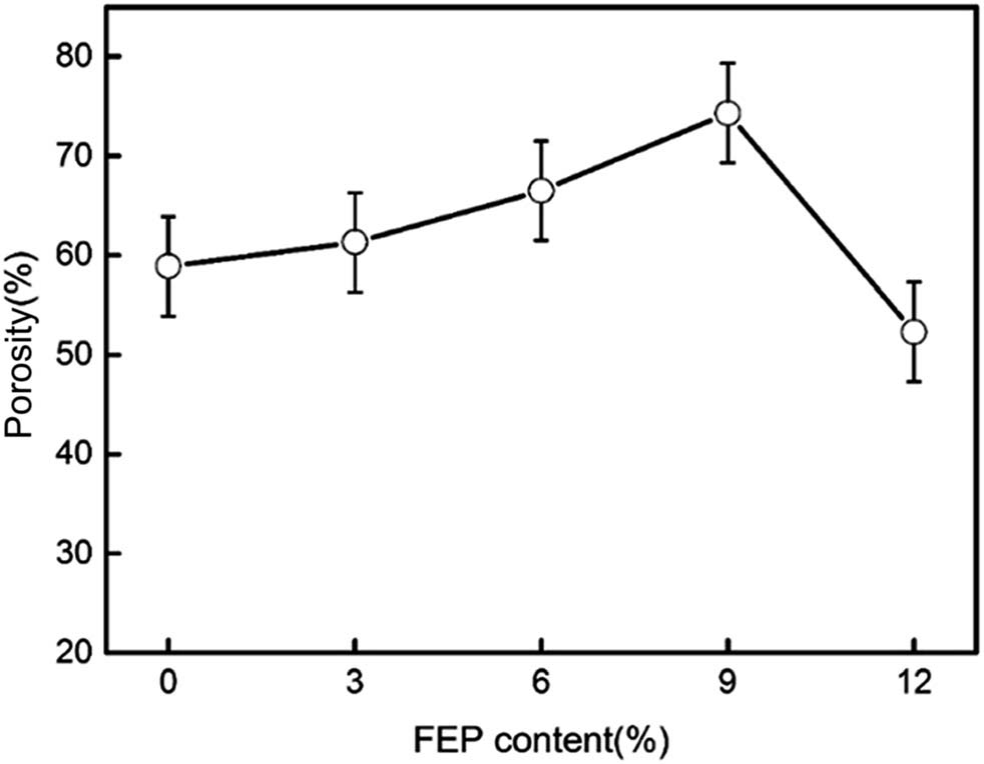
Effect of the FEP content on PSF/FEP MMMs porosity.

Porosity of the hydrophobic membrane was an important property for oil flux in membrane distillation. The porosities of PSF/FEP MMMs were measured using the gravimetric method, and the porosity results were shown in Fig. 8. It could be found that the porosity improved with increasing FEP concentration. The biggest porosity was 74.3% for M-4. That was because the membrane formed abundant and homogeneous sponge-like pore structure as shown in Fig. 2. Phase separation occurred between PSF matrix phase and FEP dispersed phase due to their different hydrophobicity. The phase separation would generate the interfacial micro voids, which improved porosity obviously. When FEP concentration was bigger than PSF concentration, the phase separation got difficult, which leaded to decreasing of porosity obviously.

The average pore size of PSF/FEP MMMs was shown in Table 2. It could be found that the pore size improved obviously with increasing FEP concentration. That was because the phase separation between PSF matrix phase and FEP dispersed phase would generate the interfacial micro voids. As shown in Fig. S1,† the pore size distribution of M-4 was narrow. The result further indicated that M-4 had homogenous porosity.

### 3.6 Flux

**Fig. 9:**
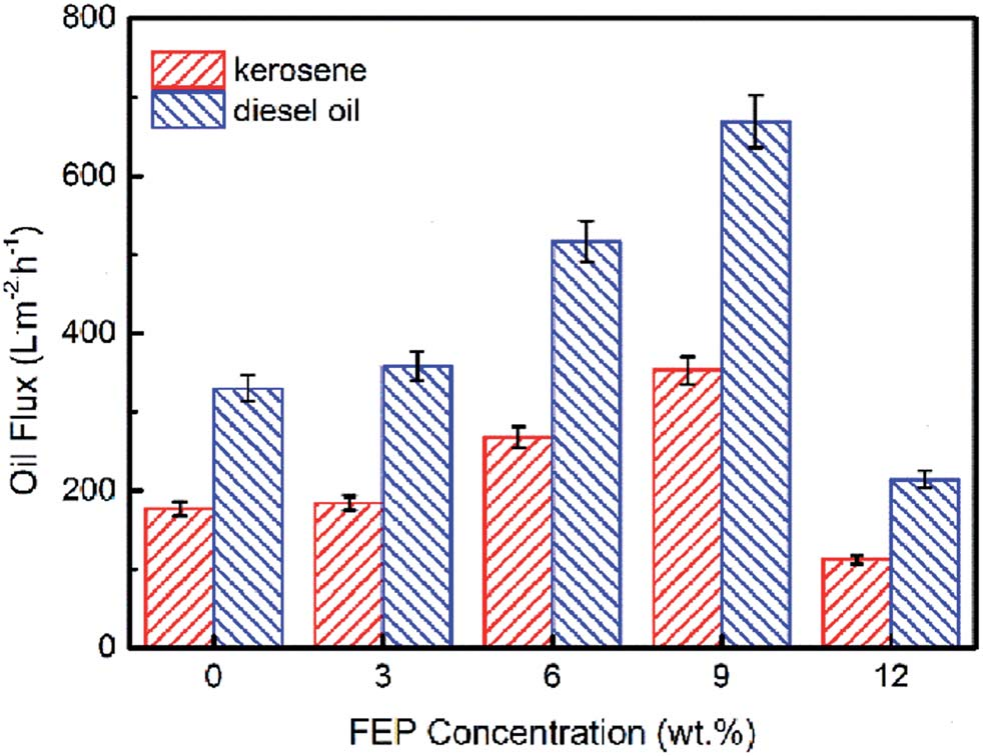
Effect of the FEP content on flux.

**Fig. 10:**
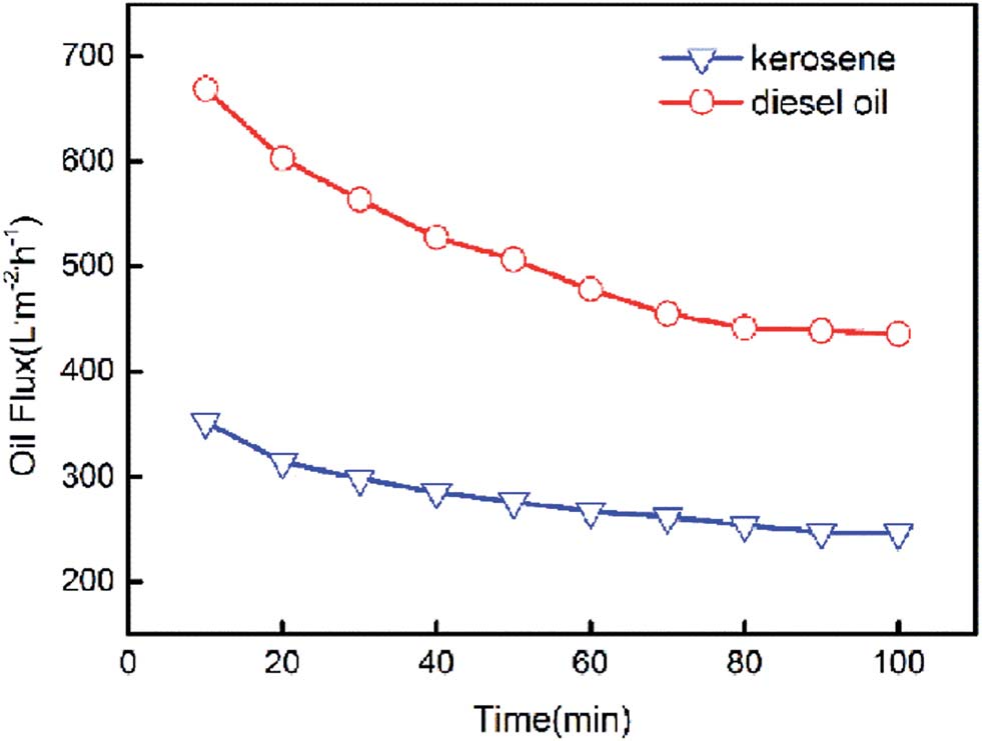
Change of oil flux with the time increase.

The pure oil fluxes of PSF/FEP MMMs for kerosene and diesel were tested and shown in Fig. 9, the maximum flux of M-4 reached as high as 352.4 L m^−2^ h^−1^ and 668.8 L m^−2^ h^−1^ for kerosene and diesel, respectively. The membrane had higher porosity and oleophilic property, when FEP concentration was 9 wt%. The flux of diesel was higher than kerosene because the viscosity of kerosene was lower. As shown in Fig. 10, with the time increasing, the fluxes of M-4 for kerosene and diesel declined. In the beginning, the pore structure was densification and the flux declined quickly. Finally, the fluxes of kerosene and diesel were stable about 246.7 L m^−2^ h^−1^ and 435.8 L m^−2^ h^−1^, respectively.

### 3.7 Separation performance

**Fig. 11:**
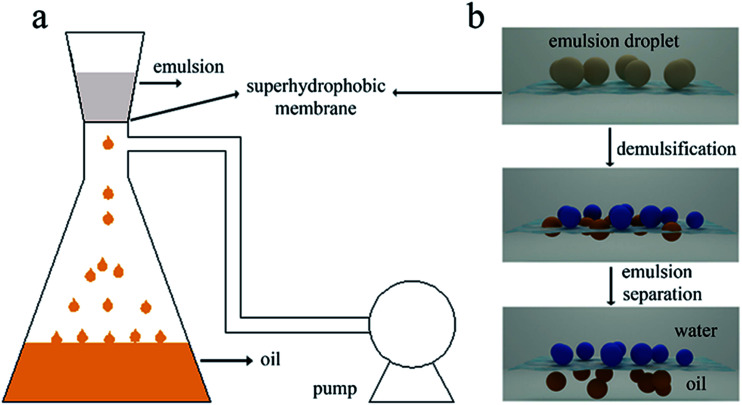
(a) The image of laboratory device for continuous water-in-oil emulsions separation. (b) The schematic diagram of emulsion separation process.

**Fig. 12:**
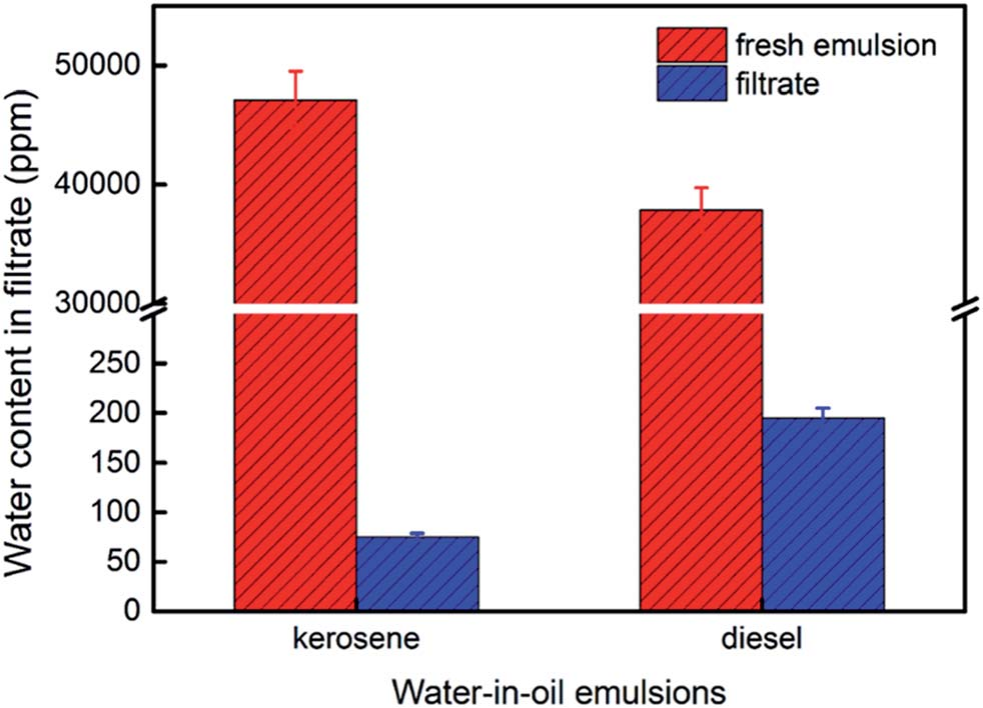
The water content in fresh water-in-oil emulsion and the filtrate.

**Fig. 13:**
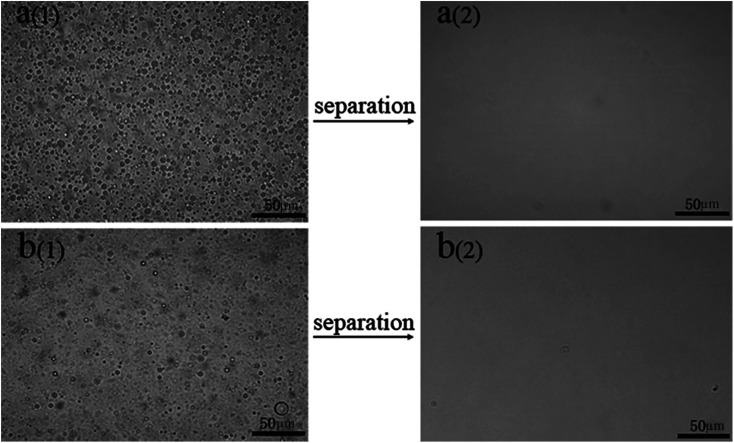
Optical microscopy images of M-4 for (a) water-in-kerosene and. (b) Water-in-diesel emulsions (1-fresh emulsions and 2-filtration).

**Fig. 14:**
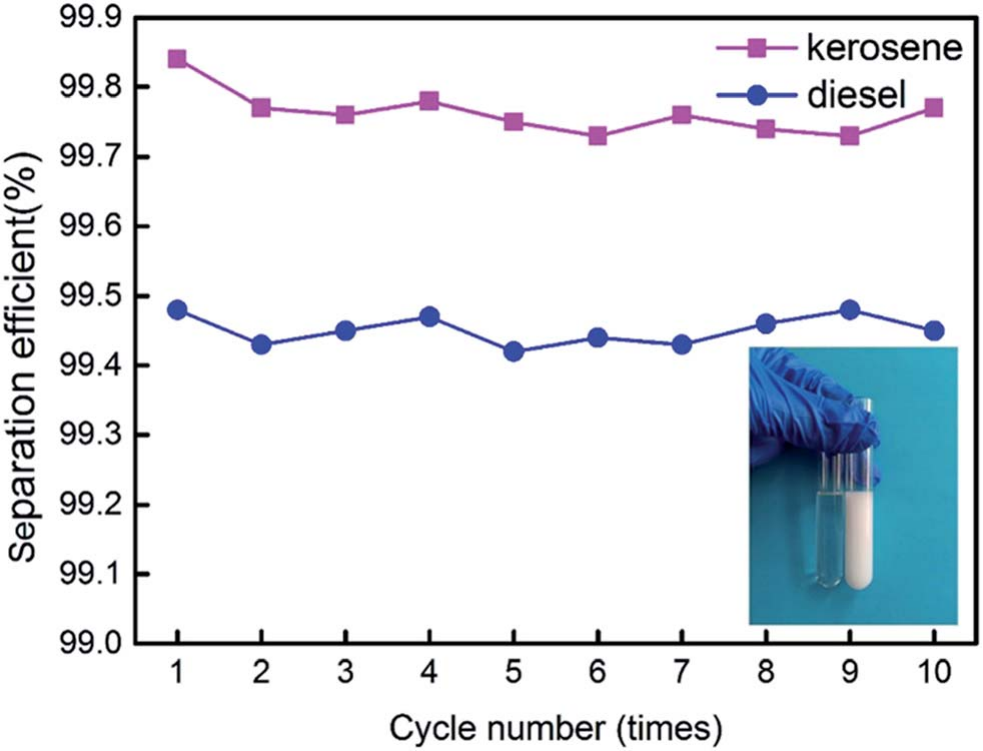
Change of the separation efficiency for M-4 with increasing cycle numbers.

We made water-in-kerosene and water-in-diesel stabilized emulsions with surfactant to conduct water-in-oil emulsion separation experiment. Fig. 11(b) showed the schematic diagram of the emulsion separation. Firstly, the water-in-oil emulsion took demulsification on the super hydrophobic membrane surface due to the different wetting performance for oil and water. Then, the oil quickly penetrated through the membrane and water was retained above. We used M-4 to conduct water-in-oil emulsion separation experiment. As shown in Fig. 12, the water content in fresh water-in-kerosene emulsion was about 47 145 ppm, and the filtrate was about 75 ppm. The water rejection rate reached 99.84%. The water content in fresh water-in-diesel emulsion was about 37 850 ppm, and the filtrate was about 195 ppm. The water rejection rate reached 99.48%. M-4 had a high water-in-oil separation precision due to the super hydrophobic surface. The optical microscopy images and droplet distribution for the separation result of water-in-kerosene and water-in-diesel emulsions were shown in Fig. 13(a) and (b), respectively. There were dense water droplets in the fresh emulsion, however not a single water droplet was observed after separation. And by comparison the picture before and after filtration, we also found that the separation effect of M-4 was very good. The separation efficiency of M-4 for water-in-oil emulsions was higher than the other reported membranes which were in different structures and materials.^42–45^ To study the separation performance further, the membrane was exposed to the water-in-oil emulsion for a long time, which would cause unavoidable membrane fouling, leading to the reduction of membrane's service life and the separation efficiency. During a separation cycle, the membrane was immersed 20 mL ethanol for 2 h and dried in air. The process was repeated ten times and the oil purity of filtrate was counted each time. As demonstrated in Fig. 14, the separation precision of M-4 was also reflected by calculating the purity of the collected filtrate in cycle 10 times. The separation efficiency for water-in-kerosene and water-in-diesel emulsions changed slightly and still maintained a relative high level of 99.79% and 99.47% after 10 cycles. It indicated that PSF/FEP MMM had an outstanding separation performance, which was in favor of using for a long time.

The comprehensive properties of PSF/FEP MMMs were shown in Table 2.

## 4. Conclusions

PSF/FEP MMMs were prepared by NIPS method successfully. SiO_2_ nanoparticles and micro-nanometer scale FEP particles fabricated unique micro- and nanoscale hierarchical structures on the membrane surface. With increasing FEP content, the structures of membrane surface changed obviously. When the content of FEP was 9 wt%, the average roughness of M-4 reached 0.712 μm. Meanwhile, the CA and the SA of M-4 was 153.3° and 6.1° respectively, showing super hydrophobicity. The fluxes of kerosene and diesel were stable about 246.7 L m^−2^ h^−1^ and 435.8 L m^−2^ h^−1^, respectively. And the separation efficiency of water-in-kerosene emulsion and water-in-diesel emulsion reached 99.79% and 99.47% after 10 cycles with an outstanding separation performance.

## Conflicts of interest

There are no conflicts of interest to declare.

## Supplementary Material

RA-008-C8RA00055G-s001
