# Supplementary figures and images for: Preparation of PSF/FEP mixed matrix membrane with super hydrophobic surface for efficient water-in-oil emulsion separation
Source: RSC Adv. 2018 Mar 14;8(18):10097–106. doi: 10.1039/c8ra00055g (PMC9078720; doi:10.1039/c8ra00055g)

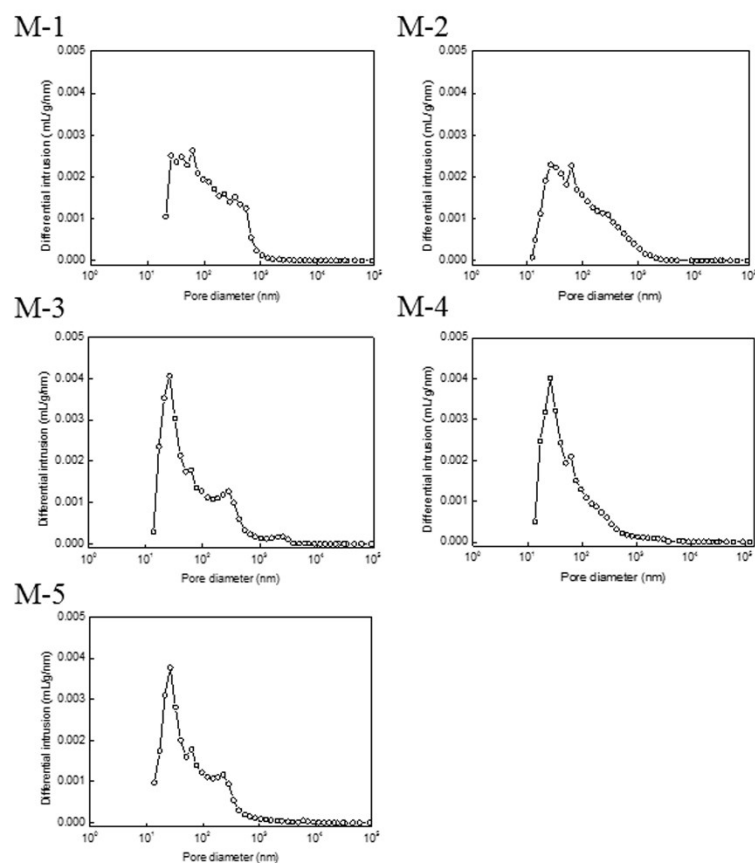

Fig. S1 The pore size distribution of the membranes.

Supplement: RA-008-C8RA00055G-s001 [file RA-008-C8RA00055G-s001.pdf]
